# Monitoring the Interaction between Thermally Induced Whey Protein and Anthocyanin by Fluorescence Quenching Spectroscopy

**DOI:** 10.3390/foods10020310

**Published:** 2021-02-03

**Authors:** Shuai Ren, M. Monica Giusti

**Affiliations:** Department of Food Science and Technology, The Ohio State University, 2015 Fyffe Rd, Columbus, OH 43210-1007, USA; ren.313@osu.edu

**Keywords:** anthocyanin color stability, anthocyanin-whey protein interaction, preheating treatment, fluorescence quenching, thermodynamic analysis

## Abstract

The color stability of anthocyanins was shown to improve with addition of whey proteins (WP). The goal of this study was to investigate the binding mechanisms of purple corn, grape and black carrot anthocyanin extracts to native and preheated WP (40–80 °C, 3.6 μM) at a pH of 3 using fluorescence quenching spectroscopy. The fluorescence spectra were collected with an excitation wavelength of 280 nm at 25 °C, 35 °C and 45 °C. The quenching data were analyzed by using the Stern–Volmer equation. The fluorescence intensity of WP decreased (up to 73%) and its λ_max_ increased (by ~5 nm) with increasing anthocyanin concentration (0–100 μM). The quenching data showed that the interaction between anthocyanin extracts and WP was a static quenching process. Thermodynamic analysis showed their binding was mainly through hydrophobic interactions. Their binding affinity was higher for preheated WP than native WP and decreased gradually with increasing preheating temperature. Black carrot anthocyanin extract had the lowest binding affinity with WP, likely due to the larger molecular structure. These results help better understand the protection mechanism of native and preheated WP on anthocyanin color stability, expanding the application of anthocyanins as food colorants that better withstand processing and storage.

## 1. Introduction

Anthocyanins (ACN), as natural food colorants, are widely studied and used frequently in the food industry to provide or improve color in food products like jams, canned foods, yogurt, juices or confectioneries, because of their bright and attractive colors from red to purple, nontoxicity and water solubility [1]. In addition to acting as food colorants, ACN have a variety of potential health benefits. Studies showed that ACN were effective antioxidants and might aid in the prevention of cardiovascular diseases, inflammation, certain cancers, diabetes and obesity [2,3]. Despite their important biological effects, ACN have limited chemical stability due to their sensitivity to different factors, such as processing conditions and storage, including other food matrices, pH, temperature, light, oxygen, metal ions, sulfur dioxide, vitamin C and enzymes [4,5]. Their limited stability constrains their commercial application as colorants in processed foods, especially those thermally processed. Therefore, it is meaningful and challengeable to find an effective way to reduce ACN loss during food processing and storage.

At present, the complexation between whey proteins (WP) and ACN is receiving increasing attention, and it is believed to occur mainly through noncovalent binding [6]. Native β-lactoglobulin (β-LG) is the one of the major proteins in WP, and is a highly-structured globular protein with 162 amino acid residues and a molecular weight of 18,400 Da [7]. It exists as a dimer at neutral pH and is folded into a calyx form by antiparallel β-sheets [8]. It has a hydrophobic interior pocket structure, called a “calyx structure”, which acts as the primary binding site for the ligands. Thus, β-LG displays a strong binding affinity for various hydrophobic and amphiphilic ligands, including phenolic compounds, fatty acids, retinol, β-carotene, phospholipids, vitamin D and some small acids [9,10,11]. Studies showed that more disordered proteins have stronger interactions with phenolic compounds than globular proteins [12]. Thus, the thermally modified WP is expected to have higher binding efficiency with ACN. Yet, limited information is available on the molecular binding interaction between ACN and WP, particularly when the WP is heated and is no longer in its native form.

Fluorescence quenching spectroscopy is useful to characterize the binding interaction and structural changes of proteins with other substances, such as polyphenols, because of its easy accessibility and accuracy [13]. Protein fluorescence is attributed to both tryptophan (Trp) and tyrosine (Tyr) residues, because they are particularly sensitive to microenvironments, especially with an excitation wavelength of 280 nm [14]. The change of the fluorescence emission intensity and maximum wavelength can identify the WP structural changes, as well as its microenvironment difference [15]. Arroyo-Maya and others demonstrated that pelargonidin quenched milk proteins fluoresce strongly due to protein ligand binding [16]. They found that cyanidin-3-glucoside quenched milk protein fluoresce strongly through hydrogen bonds and hydrophobic interactions, and that their binding affinity increased with increasing preheating temperature at pH 6.3 [6]. Chung and others found that the maximum intensity in the fluorescence emission spectra of highly denatured WP decreased with increasing ACN concentration and their interaction might be due to hydrogen bonding [17]. Quenching experiments by Stănciuc et al. showed that WP structure underwent significant conformational changes through electrostatic interaction with grape skin ACN (GSA), and GSA regularly quenched the fluorescence intensity of WP with significant red shifts in λ_max_ [18].

The purpose of this study was to investigate the interaction mechanism between selected commercial ACN-based colorants (grape juice, black carrot and purple corn ACN juice concentrates) and preheated WPs at different temperatures through fluorescence quenching spectroscopy. It was hypothesized that ACN quenches WP’s fluorescence strongly and ACN-WP binding is mainly through hydrophobic interactions. Previous studies showed that the exposure of whey protein functional groups at different levels affect the binding affinity of WP with ACN [6]. Therefore, our previous study was focused on the interaction between ACN and preheated WP, and the results showed that preheated WP could improve ACN heat and UV-light stability and better protect ACN from color degradation in the presence of ascorbic acid than native WP. Therefore, it was hypothesized that ACN-WP binding affinity was higher for preheated WP than native WP. The results could improve the understanding of thermally-induced WP-ACN interaction in the protection of ACN from degradation, as well as accelerate ACN application as food colorants in the food industry.

## 2. Materials and Methods

### 2.1. Materials and Chemicals

Grape juice ACN concentrate and black carrot ACN juice concentrates were obtained by DDW The Color House (Port Washington, WI, USA). Purple corn ACN powder was obtained from Artemis International, Inc. (Fort Wayne, IN, USA). Grass-fed WP isolate (less than 1% Non-GMO sunflower lecithin) was purchased from ProMix Nutrition (Gainesville, FL, USA). The chemicals and reagents (ACS or HPLC grade) were purchased from Fisher Scientific (Fair Lawn, NJ, USA), including methanol, acetonitrile, citric acid, hydrochloric acid (HCl), sodium phosphate dibasic (Na_2_HPO_4_), potassium chloride and sodium acetate. ACS grade ethyl acetate and formic acid were obtained from Mallinckrodt Chemicals (Bedminster Township, NJ, USA) and Honeywell (Morris Plains, NJ, USA), respectively.

### 2.2. Buffer System and Sample Preparation

The commercial WP isolate powder was dissolved in citric acid–Na_2_HPO_4_ buffer at pH 3.0 to reach a concentration of 3.6 μM. The buffer solution was prepared with 0.1 M citric acid and 0.2 M Na_2_HPO_4_ according to Dawson [19]. The WP solutions were preheated water baths (Fisher Scientific, Fair Lawn, NJ, USA) at 40 °C, 50 °C, 60 °C, 70 °C and 80 °C separately for 30 min and then quickly cooled in ice. The preheated WP solutions and their unheated counterparts (native WP) were stored at 4 °C until analysis.

Purple corn, grape and black carrot ACN extracts were semipurified through solid-phase extraction using a C-18 cartridge [20]. Ethyl acetate was used to remove less polar phenolics. The semipurified pigments were redissolved in acidified (0.01% *v*/*v* HCl) distilled water to 50 mL, and stored at −18 °C to minimize pigment degradation. The pH differential method was used to determine monomeric ACN concentration [21].

Semipurified ACN pigments were mixed with the native WP solutions at room temperature and vortexed for 20 s. The final concentration of proteins in the mixtures was 3.6 μM and the ACN concentrations were 0, 10, 20, 30, 40, 50, 60, 70, 80, 90 and 100 μM, respectively. The pH of the WP solution was not affected by the addition of small amount of ACN. To further explore the effect of WP preheating temperature on the ACN–WP interaction, 50 μM semipurified ACN pigments were mixed with different preheated WP solutions (3.6 μM, 40–80 °C) at room temperature. The fluorescence spectra were analyzed for all samples.

### 2.3. Anthocyanin Identification

Individual semipurified ACN pigment samples were then analyzed and identified by ultra-high-performance liquid chromatography (uHPLC) (Shimadzu, Columbia, MD, USA). A reverse-phase Symmetry C-18 (5 μm, 4.6 × 150 mm) column (Phenomenex, Torrance, CA, US) was used. All the extracts were filtered through a 0.22 μm syringe filter (Phenomenex, Torrance, CA, USA) before injection into the HPLC. Samples were analyzed using a flow rate of 0.2 mL/min. The mobile phase consisted of solvent (A) 4.5% (*v*/*v*) formic acid and solvent (B) 100% acetonitrile. For black carrot ACN, the linear gradient used in the analysis was from 2–8% B 1 min–6 min, 8% B 6–10 min, 8–20% B 10 min–13 min, 20–40% B 13 min–13.5 min, 40% B 13.5–14 min and 40–2% B 14–14.5 min. For purple corn ACN, the linear gradient used was from 2–20% B 1 min–15 min, 20–40% B 15–15.5 min, 40% B 15.5 min–16 min and 40–2% B 16–16.5 min. For grape ACN, the linear gradient was from 2–6% B 1 min–2 min, 6–10% B 2–10 min, 10–40% B 10 min–24 min, 40% B 24 min–26 min and 40–2% B 26–27 min. Elution of ACN and all phenolics were monitored at 520 nm and 280 nm respectively, and the whole spectrum (260–700 nm) was also collected. Total ion scans and selected ion monitoring were conducted. Mass to charge ratios of 271, 287, 303, 301, 317 and 331 corresponded to the most six common ACN aglycones.

### 2.4. Fluorescence Spectroscopy

For native WP, thermally-induced WP and WP–ACN mixtures, 250 μL of each sample was aliquoted to Greiner black 96-well plates, and the spectra were analyzed by using a SpectraMax M2 Microplate Reader (Molecular Devices, Sunnyvale, CA, USA). The fluorescence was measured at a fixed WP concentration (3.6 μM) in the presence of various concentrations of ACN rich extracts (0~100 μM). The emission spectra were individually recorded from 360 to 450 nm, with an excitation wavelength of 280 nm at 25 °C, 35 °C and 45 °C [16]. All tests were done in triplicate.

### 2.5. Fluorescence Quenching Analysis

The fluorescence quenching mechanism can be described using the Stern–Volmer Equation (1):

F_0_/F = 1 + K_SV_ [Q] = 1 + K_q_τ_0_ [Q], (1)

where F_0_ and F represent the fluorescence intensities of proteins alone and in the presence of a given concentration of ACN (quencher), respectively, Q is the concentration of free ACN and K_sv_ is the Stern–Volmer constant, which is given by the result of the quenching bimolecular rate constant K_sv_ = K_q_τ_0_, where K_q_ is the bimolecular quenching constant and τ_0_ is the lifetime of the fluorophore being quenched and equals 10^−8^ s [22]. If K_q_ was much higher than the limiting diffusion rate constant of the biomolecules (2 × 10^10^ M^−1^s^−1^), it indicated that the WP and ACN interaction was mainly static quenching. For static quenching, the binding constant (K_s_) was calculated using nonlinear least-squares fitting of the experimental data, as shown in Equation (2) [23]:

log[(F_0_ − F)/F] = log K_s_ + m log[Q], (2)

where m represents the kinetic reaction order (molecularity in Q). M measures the number of Q molecules that interact simultaneously with each site and does not express the number of independent and equivalent binding sites [23].

### 2.6. Thermodynamic Analysis

The energetics of protein–ligand equilibrium can be characterized by the Gibbs free energy (∆Go), the binding enthalpy (∆Ho) and entropy (∆So). These parameters were used to determine the type of interaction between biomacromolecules and small molecules [24]. The ∆Go was calculated by Equation (3). The ∆Ho and ∆So were achieved with linear fitting using the van’t Hoff Equations (4) and (5) [25]:

∆Go = −RTlnK_s_, (3)

lnK_s_ = (−∆Ho/R)(1/T) + (∆So/R), (4)

∆Go = ∆Ho − T∆So,(5)

where T is the absolute temperature (K), K_s_ is the binding constant associated with temperatures and R is the gas constant (8.314 J mol^−1^ K^−1^).

### 2.7. Statistical Analysis

All the analyses were done in triplicate and evaluated by their means and standard deviations. Statistical differences between two groups were evaluated using the Tukey posthoc test by SPSS (IBM, Armonk, New York, NY, USA). A probability value of *p* < 0.05 was considered to be significant. The best-fit values for binding parameters were calculated by applying nonlinear least-squares regression using Microsoft Excel (Microsoft Corporation, One Microsoft Way, Redmond, Washington, DC, USA).

## 3. Results

### 3.1. Analysis of Fluorescence Spectra of Whey Protein in the Presence of Anthocyanins

Purple corn, grape and black carrot were selected as three ACN sources in this study. Their compositions were analyzed by ultra-high-performance liquid chromatography (uHPLC). The purities for grape, black carrot and purple corn ACN in the ACN extracts were 84.50%, 88.63% and 81.72%. Grape ACN extract mainly contains delphinidin (Dp)-3,5-diglucoside (2.64%), cyanidin (Cy)-3,5-diglucoside (1.16%), petunidin (Pt)-3,5-diglucoside (5.70%), peonidin (Pe) -3,5-diglucoside (19.12%), malvidin (Mv)-3,5-diglucoside (37.77%), Mv-3-glucoside (2.62%), Pt-3-coumaroyl-5-diglucoside (1.84%), Pe-3-coumaroyl-5-diglucoside (1.50%) and Mv-3-coumaroyl-5-diglucoside (9.54%). Most anthocyanin were glycosylated by two glucoses. The main anthocyanins were mal-3,5-diglucoside and peo-3,5-diglucoside and their acylation counterparts. Black carrot ACN was mainly acylated, such as Cy-3-xylgalactoside (12.55%), Cy-3-xylosyl-(sinapoyl-glucosyl)-galactoside (7.14%), Cy-3-xylosyl-(feruloyl-glucosyl)-galactoside (35.35%), Cy-3-xylosyl-(coumaroyl-glucosyl)-galactoside (11.54%), Pe-3-galalactoside (1.68%) and Pe-3-xylosyl-(sinapoyl-glucosyl)-galactoside (3.37%). All the ACN were glycosylated by one galactose. The main ACN were cyanidin-based (~68% of total anthocyanins) and most of them were acylated (~57% of total ACN). Compared with the other two ACN, black carrot ACN showed a bigger molecular structure, because most of the ACN had two or three sugars. Purple corn ACN included Cy-3-glucoside (20.64%), Pe-3-glucoside (4.14%), Pe-3-glucoside (8.27%), Cy-3-(6″-malonylglucoside) (4.56%) and Pe-3-(6″-malonylglucoside) (2.90%). All peaks were glycosylated by one glucose and the main ACN were Cy-3-glucoside, Pe-3-glucoside and their malonic acid acylation counterparts.

**Figure 1:**
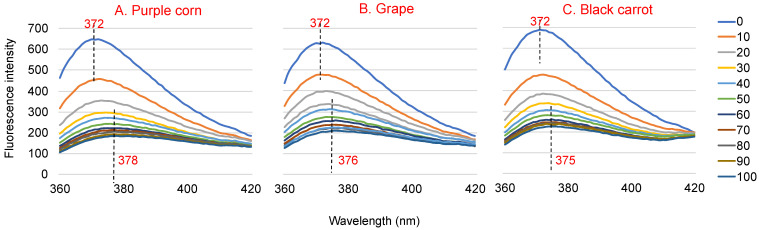
Fluorescence spectra of 3.6 μM native whey protein in the presence of 0~100 μM purple corn (**A**), grape (**B**) and black carrot (**C**) anthocyanin-rich extracts at an excitation wavelength of 280 nm at 25 °C and pH 3.

Addition of ACN-rich extracts to WP solutions resulted in changes to the WP fluorescence spectra, both in fluorescence intensity and λ_max_. ACN-rich extracts may have some fluorescence, but their intensities were negligible compared to that of WP [26]. Therefore, the change in fluorescence can be attributed to the interaction between the extract and the WP. Figure 1 shows the fluorescence emission spectra of 3.6 μM WP with the different concentrations of ACN rich extracts (0~100 μM) at an excitation wavelength of 280 nm. The λ_max_ of WP was around 372 nm. The fluorescence intensity of the WP–ACN solutions decreased with increasing ACN concentration, which indicated ACN had a concentration-dependent quenching effect on WP fluorescence. When ACN concentration increased to 100 μM, the fluorescence intensity decreased by about 68% for grape and black carrot ACN extracts and 73% for purple corn ACN extract. The λ_max_ of WP showed a red shift from 372 nm to 378 nm with increasing ACN concentration (Figure 1). This indicated that the microenvironment of WP became more hydrophilic after the addition of ACN [27]. A similar finding was also shown in a previous study, where the fluorescence intensity of β-LG decreased and the λ_max_ of β-LG showed a red shift with increasing malvidin-3-glucoside concentration [28].

### 3.2. Fluorescence Quenching Study of Anthocyanin-Whey Protein Interaction

**Figure 2:**
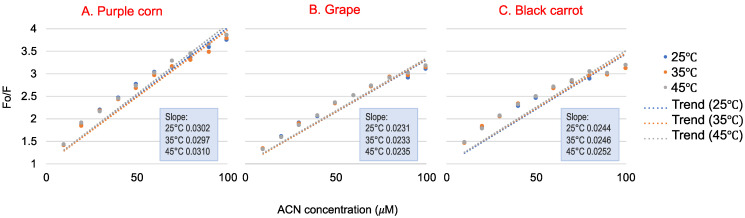
The Stern–Volmer plots for the quenching of whey protein by purple corn (**A**), grape (**B**) and black carrot (**C**) anthocyanin-rich extracts at 25 °C, 35 °C and 45 °C (used for K_sv_ and K_q_ calculations). Data presented are means of three replications. Standard deviations for all data points are less than 0.1.

Figure 2 shows the Stern–Volmer plot of WP fluorescence quenched by different concentrations of ACN at 25 °C, 35 °C and 45 °C, and relative quenching parameters were calculated; these are shown in Table 1. The K_q_ parameters were used to determine the quenching process. Fluorescence quenching can be classified into static quenching and dynamic quenching [29]. The two different quenching processes are reported to be influenced by the temperature of the environment [30]. Static quenching is controlled by the formation of a complex between the fluorophore and the quencher. Therefore, the static quenching constant decreases with temperature, because the stability of complex may be affected by the increased temperature [30]. Dynamic quenching is caused by diffusion and collision encounters. Therefore, the dynamic quenching constant will increase at higher temperatures due to higher diffusion and collision [31]. Studies indicated that the linear range in the Stern–Volmer plot could be used to determine whether the quenching mechanism was static or dynamic [32]. The K_q_ values obtained from the three reactions were much higher than the limiting diffusion rate constant of the biomolecules (2 × 10^10^ M^−1^s^−1^), indicating that all three ACN-rich extracts could quench the WP fluorescence through static quenching, which was induced mainly by the formation of ACN-WP complex [29]. The K_q_ value had a slight, but not significant, increase as temperature increased, so it did not provide clear evidence of dynamic quenching between ACN and WP. Further, for purple corn ACN, the K_q_ value at 35 °C was lower than that at 25 °C, which again suggested that ACN–WP was not dynamic quenching. On the other hand, the data in Figure 2 seemed a little nonlinear, which might be explained by the fact that the samples in this study were a mixture of ACN extracts rather than an ACN isolate. The phenolics or other compounds in the extracts might interfere with the ACN–WP quenching, and resulting in a more complex quenching process. A similar result was also found by Khalifa et al., showing that a static and heat-stable binding occurred between WP and mulberry ACN extract, leading to WP size and secondary structure changes [33].

**Figure 3:**
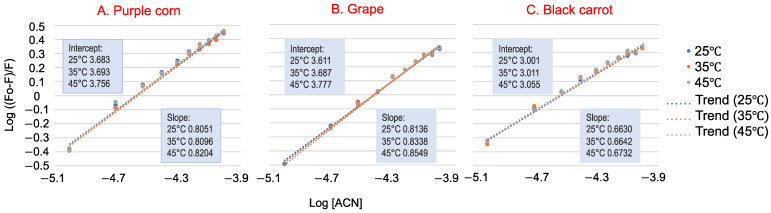
The double logarithm regression plots of log [(F_0_ − F)/F] versus log [ACN] (purple corn (**A**), grape (**B**) and black carrot (**C**)) of whey protein at 25 °C, 35 °C and 45 °C (used for K_s_ calculation). Data presented are means of three replications. Standard deviations for all data points are less than 0.02.

For the static quenching process, the double logarithm regression curves of log [(F_0_ − F)/F] versus log [ACN] are shown in Figure 3, and the binding constants K_s_ are listed in Table 1. The K_s_ for all three ACN–WP solutions were in the order of 10^3^, indicating that ACN-rich extracts had a strong binding affinity toward WP. The K_s_ values increased with increasing temperature, so their binding reaction was endothermic [24]. Black carrot ACN extract had a much smaller binding affinity with WP (K_s_ ~ 1 × 10^3^ M^−1^) than purple corn and grape ACN extracts (K_s_ ~ 5 × 10^3^ M^−1^). This could be explained by their different ACN molecular structures. According to our previous study, black carrot ACN were mainly acylated with two or three glycosylations. Thus, the large molecular structure of black carrot ACN extract inhibited interaction with WP, resulting in lower binding affinity. Further, purple corn and grape ACN extracts exhibited lower ACN purity than black carrot ACN extract, meaning that other compounds other than ACN that remain in the extract, like phenolics, also bind with WP.

### 3.3. Thermodynamic Analysis between Whey Protein and Anthocyanin

The thermodynamic parameters associated with the WP–ACN interactions are shown in Table 1. The signs and magnitudes of thermodynamic symbols were used to determine the different binding force types [34]. There are four main types of noncovalent binding forces involved in the interaction between molecules, namely, hydrophobic interaction, hydrogen bonding, electrostatic interactions and van der Waals force [24]. Typically, ∆Ho > 0, ∆So < 0 is related to electrostatic and hydrophobic force, ∆Ho > 0, ∆So > 0 identifies hydrophobic force, ∆Ho < 0, ∆So < 0 suggests van der Waals forces or hydrogen bonding, and ∆Ho ~ 0, ∆So > 0 implies electrostatic force [34]. However, protein association reactions are very complex and thermodynamic parameters are affected by many factors and cannot be decided by a simple rule [34]. As shown in Table 1, the ∆Go values for all three ACN-rich extracts were negative, which indicated that the binding process of WP and ACN-rich extract was spontaneous [35]. For purple corn and black carrot ACN extracts, ∆Ho > 0 and ∆So > 0 suggested that hydrophobic interactions were the dominant binding force for the formation of the ACN–WP complex. The ∆Ho > 0 also indicated that the reactions were endothermic. For grape ACN extract, ∆Ho < 0 and ∆So > 0 suggested that electrostatic interactions might play a major role in the ACN–WP binding process. However, studies showed that in classic electrostatic interactions, the negative ∆Ho is very small (around zero) [34,36]. In this study, the ∆Ho value for grape ACN–WP complex was much lower than 0. Therefore, the grape ACN–WP interaction may not be caused by electrostatic interactions. On the other hand, a positive ∆So was observed in the grape ACN–WP complex, which is also expected in hydrophobic interactions. Therefore, the grape ACN–WP interaction was more likely to be induced by hydrophobic interactions rather than electrostatic interactions. A similar study also showed that the grape skin ACN extracts and β-lactoglobulin complex interacted mainly through hydrophobic interaction [28].

### 3.4. The Effect of Preheating Temperature on the Fluorescence Spectra of Whey Protein in the Presence of Anthocyanins

**Figure 4:**
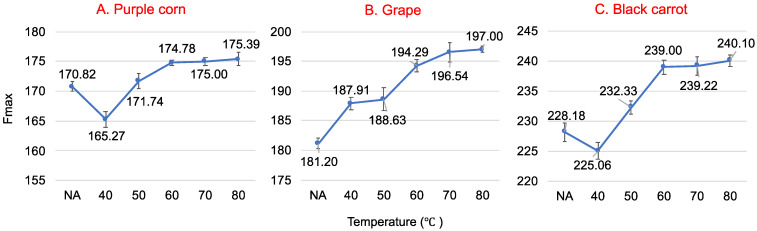
Fluorescence maximum intensity change of native and preheated whey protein (40 °C, 50 °C, 60 °C, 70 °C and 80 °C) at 25 °C in the presence of 50 µM purple corn (**A**), grape (**B**) and black carrot (**C**) anthocyanin-rich extracts at an excitation wavelength of 280 nm at pH 3.

Protein structure changes, such as denaturation and aggregation, can be observed through emission spectra changes [37]. WP after heat treatment at different temperatures presents conformational changes, which induces the change of its intrinsic fluorescence. The fluorescence maximum intensity change of native and preheated WP at 25 °C in the presence of ACN (50 μM) at pH 3 are shown in Figure 4. The maximum fluorescence intensity F_max_ increased significantly (*p* < 0.05) as the temperature increased from 40 °C to 60 °C. This may have occurred because Trp was exposed to heat treatment. WP disulfide-linked aggregation decreased the exposure of Trp [38] as temperature increased, thus decreasing the quenching effect. No significant difference was observed (*p* > 0.05) for F_max_ when WP was heated between 60 °C and 80 °C. This could be due to the irreversible Tanford-like transition after WP was heated for 30 min at high temperatures [39]. The breaking of the disulfide bonds and the exposure of all the previously buried Trp increased ACN quenching to the highest level [38]. Moreover, the conversion of regular structures (α-helices, β-sheets) of WP into more unfolded structures also resulted in achieving the highest F_max_ [39].

The quenching parameters for native and preheated WP fluorescence quenched by various concentrations of ACN extract at 25 °C are shown in Table 2. The K_q_ values (>2 × 10^10^ M^−1^s^−1^) indicated that ACN-rich extracts effectively quenched the intrinsic fluorescence of WP through static quenching due to the formation of WP–ACN complexes [28]. The binding constant K_s_ of native WP at 25 °C was lower than that of preheated WP, indicating a greater affinity of ACN for preheated WP. When the preheating temperatures increased, K_s_ decreased slightly but not significantly (*p* > 0.05). This might be due to the increased WP aggregation [6].

## 4. Conclusions

The WP’s fluorescence was quenched effectively by ACN-rich extracts. The interaction between ACN-rich extracts and WP decreased the fluorescence intensity of WP and increased its λ_max_. The interaction between ACN-rich extracts and WP was mainly a static quenching process and occurred mainly via hydrophobic forces. Black carrot had the lowest binding affinity with WP, likely due to its large molecular structure. The binding affinity of WP and ACN-rich extracts was affected by the different WP preheating temperatures, likely due to changes in WP structural conformations. The ACN binding affinity was higher for preheated WP than native WP and decreased gradually with increasing WP preheating temperature, most likely due to protein aggregation. These results may improve our understanding of how thermally induced WP protect ACN from color degradation, and therefore help broaden the range of applications of ACN as food colorants in the food industry.

## Figures and Tables

**Table 1:** The Stern–Volmer constants, quenching constants, binding constants and thermodynamic parameters for purple corn, grape and black carrot anthocyanin-rich extracts binding to whey protein at 25 °C, 35 °C and 45 °C (pH 3).

Parameters		T (°C)		
		25	35	45
Purple corn	K_sv_ (×10^4^ M^−1^)	3.02 ± 0.07	2.97 ± 0.13	3.1 ± 0.05
	K_q_ (×10^12^ M^−1^s^−1^)	3.02 ± 0.07	2.97 ± 0.13	3.1 ± 0.05
	K_s_ (×10^3^ M^−1^)	4.82 ± 0.15	4.94 ± 0.28	5.70 ± 0.19
	ΔH^o^ (×10^3^ J mol^−1^)	6.52		
	ΔG^o^ (×10^4^ J mol^−1^)	−2.10	−2.18	−2.29
	ΔS^o^ (J mol^−1^ K^−1^)	92.40	70.71	71.89
Grape	K_sv_ (×10^4^ M^−1^)	2.31 ± 0.07	2.33 ± 0.04	2.35 ± 0.07
	K_q_ (×10^12^ M^−1^s^−1^)	2.31 ± 0.07	2.33 ± 0.04	2.35 ± 0.07
	K_s_ (×10^3^ M^−1^)	4.09 ± 0.78	4.86 ± 0.79	5.99 ± 1.23
	ΔH^o^ (×10^3^ J mol^−1^)	−6.49		
	ΔG^o^ (×10^4^ J mol^−1^)	−2.06	−2.10	−2.15
	ΔS^o^ (J mol^−1^ K^−1^)	47.36	68.29	67.77
Black carrot	K_sv_ (×10^4^ M^−1^)	2.44 ± 0.11	2.46 ± 0.04	2.52 ± 0.03
	K_q_ (×10^12^ M^−1^s^−1^)	2.44 ± 0.11	2.46 ± 0.04	2.52 ± 0.03
	K_s_ (×10^3^ M^−1^)	1.00 ± 0.21	1.03 ± 0.12	1.14 ± 0.18
	ΔH^o^ (×10^3^ J mol^−1^)	4.90		
	ΔG^o^ (×10^4^ J mol^−1^)	−1.71	−1.78	−1.86
	ΔS^o^ (J mol^−1^ K^−1^)	73.89	57.64	58.49

**Table 2:** The Stern–Volmer constants, quenching constants and binding constants for purple corn, grape and black carrot anthocyanin-rich extracts binding to native and preheated whey protein at 25 °C (pH 3) at an excitation wavelength of 280 nm.

Parameters		Preheat T (°C)					
		Native	40	50	60	70	80
Purple corn	K_sv_ (×10^4^ M^−1^)	1.67 ± 0.09	1.69 ± 0.02	1.71 ± 0.09	1.82 ± 0.04	1.86 ± 0.07	1.98 ± 0.06
	K_q_ (×10^12^ M^−1^s^−1^)	1.67 ± 0.09	1.69 ± 0.02	1.71 ± 0.09	1.82 ± 0.04	1.86 ± 0.07	1.98 ± 0.06
	K_s_ (×10^2^ M^−1^)	3.33 ± 0.93	4.78 ± 0.95	4.75 ± 0.33	4.65 ± 0.71	4.08 ± 0.40	3.77 ± 0.75
Grape	K_sv_ (×10^4^ M^−1^)	1.44 ± 0.17	1.28 ± 0.09	1.29 ± 0.06	1.31 ± 0.07	1.60 ± 0.06	1.61 ± 0.03
	K_q_ (×10^12^ M^−1^s^−1^)	1.44 ± 0.17	1.28 ± 0.09	1.29 ± 0.06	1.31 ± 0.07	1.60 ± 0.06	1.61 ± 0.03
	K_s_ (×10^2^ M^−1^)	0.89 ± 0.26	1.32 ± 0.10	1.31 ± 0.03	1.25 ± 0.07	1.08 ± 0.53	0.97 ± 0.17
Black carrot	K_sv_ (×10^3^ M^−1^)	6.7 ± 0.49	6.7 ± 0.57	6.7 ± 0.78	6.8 ± 0.50	7.8 ± 0.99	8.8 ± 0.71
	K_q_ (×10^11^ M^−1^s^−1^)	6.7 ± 0.49	6.7 ± 0.57	6.7 ± 0.78	6.8 ± 0.50	7.8 ± 0.99	8.8 ± 0.71
	K_s_ (×10^2^ M^−1^)	1.07 ± 0.49	2.66 ± 0.23	2.54 ± 0.84	2.00 ± 0.15	1.42 ± 0.72	1.25 ± 0.77

## Data Availability

Not applicable.
